# Medical and Physical Intelligence

**Published:** 1803-05-01

**Authors:** 


### MEDICAL AND PHYSICAL INTELLIGENCE.

### [ FOREIGN AND DOMESTIC. ]

Dr. ERDMAN, extolls highly the singular efficacy of the spongia usta in all sorts of glandular diseases. The use it has in bronchocele and struma, induced him to try it also in other diseases of the glands, which appear to be analogous in their origin and proximate cause with the former. He in the first place mentions his mode of giving this remedy in struma, which is the following:

℞. Spong. ust. unc. ß. Sacch. alb. unc. jß. M. f. pulv. s.; a tea-spoon full to be taken dry early in the morning and at night.

At the same time he orders a stiff collar to be worn and to keep the neck warm. In this manner the tumor generally disappears within two months, but it is advisable to wear the collar for some time after.

Dr. E. having observed, that the hooping cough particularly attacks scrophulous and rickety children, and that often immediately after obstinate hooping coughs, glandular tumors and scrophulous atrophiæ supervene, he conceived the opinion, that the nature of this disease might originate from the miasmata being deposited in the small glandules of the trachea and bronchiæ, by which they are obstructed, and thus the violent irritation and spasm are produced, which so often suffocate children. In consequence of this conjecture, Dr. E. employed the spongia usta in that affection, and he had the satisfaction to observe the most surprizing advantages result from the continued use of that remedy. The disease diminished by degrees and was entirely removed by the sole use of that remedy, except in cases where an hectic fever had already taken place.

It is farther an almost infallible remedy in chronic hoarseness, particularly when caused by a catarrhous matter, which is deposited on the glandules of the larynx and trachea. In this complaint it proved of great service, even when blisters, resolvents, and other remedies had been employed without success.

The spongia usta is likewise very servicable in metastases on the glands, occasioned by the measles and the scarlet fever.

In the rickets this remedy was used with advantage, combined with different aromata, cortex cinnamomi, cortex aurant. caryophyll. &c.

Obstinate cardialgies seem in some cases to be owing to an obstruction or schirrus of the pancreas, at least Dr. E. conformable to this idea, gave the spongia usta with great success in several cases of the above disease; the dose was one scruple or half a drachm a day; day. Sometimes he prescribed a tea of valerian, chamomile flowers and orange leaves to be used at the same time, which, when employed alone, had never proved efficacious. By this method Dr. Erdman cured several cases of the most inveterate cardialgy, for which the whole series of antispasmodic remedies had been tried without effect. He concludes his observations, with remarking that he never saw any ill consequence arise from the application of that remedy, and he even gave it to persons who had been a short time before spitting blood, and who had a shortness of breath, without its ever having done them any harm.

---

M. I. P. TERRAS, Surgeon of Geneva, recommends the administration of mercurial ointment internally as very advantageous in the cure of the venereal disease. This mode of administering mercury he found had been already employed by Dr. Frize, of Berlin; he recommends the ointment to be prepared with care and composed of equal parts of lard and mercury, which he says should be prepared at least six months before it is used, in order to give time to the sebaceous acid to reduce the mercury to a saline state. M. T. gives several cases to prove the efficacy of this preparation, but which contain nothing of interest or worthy of remark. M. Sedillot, one of the Editors of the Recueil Periodique, of the Medical Society, follows up these observations by recommending what he calls a mercurial soap, instead of the preparation recommended by M. Terras, which he says had been used long since, as well by himself as his brother in the Hospital of the Beutre, near Paris; a sense of weight and other inconveniences, which he observed from the use of mercurial ointment internally, induced him to try the following form: ℞. Soap, 2 oz. double merc. ointment, 3 oz. starch, 1 oz. The whole to be made into a mass, and divided into pills of six grains each; the dose is from three to twelve pills in the day. This medicine, he says, is well fitted to remove glandular swellings or tophous concretions from a venereal cause. It is in these cases M. Sedillot's brother has employed it for more than twenty years, and he is convinced that of all the mercurial preparations this is the most powerful. He allows however, that mercurial frictions are sometimes necessary, as well as solution of corrosive sublimate, decoction of the woods, &c.

---

At a late meeting of the Galvanic Society of Paris, M. M. Nauche and Pagot Laporet communicated a new Galvanic fact. They observed that frogs, in a temperature of 10 deg. below 0 of Reaumur, when submitted to the reiterated contact of the conductor communicating with the copper pole, the devellopement of a whitish mucus fluid was very abundantly produced from the liver, lungs, and particularly from the nerves of the heart; that it was but in small quantity from the muscles, intestines, and hardly any from the integuments; while the conductor communicating with the the zinc pole, far from producing the same effect, rather appeared to counteract it.

---

Mr. Astley Cooper, Surgeon of Guy's Hospital, will publish in the course of the summer, a Treatise on the Anatomy and Surgical Treatment of Hernia, illustrated with plates of the size of Nature, principally taken from cases that have been admitted into St. Thomas's and Guy's Hospitals. The work will be divided into three parts. The first will contain the description of every species of inguinal hernia, one of which is its passage on the inner side of the epigastric artery; in the second, the subjects of hernia tunicæ vaginalis, and femoral hernia, will be treated of; and the third part will contain hernia of the navel, ischiatic notch, foramen ovale, and some hitherto undescribed varieties.

---

Mr. Wilkinson, of Soho-square, whose scientific Lectures on Galvanism have deservedly attracted so much notice, will speedily publish his promised History of that interesting science, drawn chiefly from Citizen Sue's work, to which he proposes to add a familiar introduction to the practice of Galvanism, a description of the various apparatus, and an account of the last experiments.

